# Point-of-care optical spectroscopy platform and ratio-metric algorithms for rapid and systematic functional characterization of biological models *in vivo*

**DOI:** 10.1117/1.JBO.29.12.125002

**Published:** 2024-12-31

**Authors:** Md Zahid Hasan, Jing Yan, Caigang Zhu

**Affiliations:** University of Kentucky, Department of Biomedical Engineering, Lexington, Kentucky, United States

**Keywords:** optical spectroscopy, tumor metabolism, vasculature, radiation therapy, head and neck cancer

## Abstract

**Significance:**

Cellular metabolism is highly dynamic and strongly influenced by its local vascular microenvironment, gaining a systems-level view of cell metabolism *in vivo* is essential in understanding many critical biomedical problems in a broad range of disciplines. However, very few existing metabolic tools can quantify the major metabolic and vascular parameters together in biological tissues *in vivo* with easy access.

**Aim:**

We aim to fill the technical gap by demonstrating a point-of-care, easy-to-use, easy-to-access, rapid, systematic optical spectroscopy platform for metabolic and vascular characterizations on biological models *in vivo* to enable scientific discoveries to translate more efficiently to clinical interventions.

**Approach:**

We developed a highly portable optical spectroscopy platform with a tumor-sensitive fiber probe and easy-to-use spectroscopic algorithms for multi-parametric metabolic and vascular characterizations of biological tissues *in vivo*. We then demonstrated our optical spectroscopy on tissue-mimicking phantoms, human subjects, and small *in vivo* tumor models. We also validated the proposed easy-to-use algorithms with the Monte Carlo inversion models for accurate and rapid spectroscopic data processing.

**Results:**

Our tissue-mimicking phantom, human subjects, and *in vivo* animal studies showed that our portable optical spectroscopy along with the new spectroscopic algorithms could quantify the major metabolic and vascular parameters on biological tissues with a high accuracy. We also captured the highly diverse metabolic and vascular phenotypes of head and neck tumors with different radiation sensitivities.

**Conclusions:**

Our highly portable optical spectroscopy platform along with easy-to-use spectroscopic algorithms will provide an easy-to-access way for rapid and systematic characterizations of biological tissue metabolism and vascular microenvironment *in vivo*, which may significantly advance translational cancer research in the future.

## 1 Introduction

Cellular metabolism is highly dynamic and strongly influenced by its local vascular microenvironment; gaining a systems-level view of cell metabolism and vasculature *in vivo* is essential in understanding many critical biomedical problems in a broad range of disciplines.^1^^–^^4^ For example, many types of human tumors can flexibly switch between glycolysis and mitochondrial metabolism under a range of oxygen conditions, which renders some therapies ineffective.^5^^–^^7^ Therefore, capturing both metabolism and vascular microenvironment alterations will be critical in understanding tumor treatment resistance and recurrence mechanisms. Although several tools with a variety of length scales were developed to measure the metabolic alterations in tumor cells or tissues, they cannot detect the transient and dynamic metabolic changes *in vivo* with easy access and they have a variety of practical and scientific limitations. Seahorse assay measures the oxygen consumption rate (OCR) and extracellular acidification rates (ECAR) of *in vitro* cells to report cell mitochondrial respiration and glycolysis indirectly.^8^ Metabolomics can simultaneously screen many metabolites and map the metabolic networks from *in vitro* cells to *ex vivo* tissues, but it is destructive.^9^ Immunohistochemistry (IHC) can quantify vascular endothelial markers (CD31),^10^ hypoxia (pimonidazole),^11^ glucose transporters (GLUT-1),^12^ and mitochondrial biogenesis (PGC-1α)^13^ but only for *ex vivo* tissues or *in vitro* cells. Positron emission tomography (PET) has been widely used for cancer screening *in vivo* but primarily for quantifying glucose uptake.^14^ Magnetic resonance spectral imaging (MRSI) can report on mitochondrial metabolism and glycolysis via ^31^P or ^13^C *in vivo*,^15^^,^^16^ whereas blood oxygen level-dependent magnetic resonance imaging (MRI)^17^ and dynamic contrast-enhanced MRI^18^ enable vascular imaging. Although powerful, MRI techniques have relatively low sensitivity. Unfortunately, none of the existing tools discussed above can quantify glycolysis, mitochondrial function, and vascular microenvironment together *in vivo*. Furthermore, most of them are (1) housed in core facilities that require transporting samples or animals to designated locations, (2) expensive, (3) time-consuming, and (4) expertise-dependent due to special sample preparation and complicated data processing. These factors all limit their user access to biomedical research. It is significant to break the limitations of conventional equipment and develop new tools capable of quantifying tissue metabolism and vasculature together *in vivo.* To maximize access to biomedical research across research labs, it is critical to develop new metabolic tools with high portability and low-cost footprints.^19^^–^^22^

Optical spectroscopy techniques have great potential to provide point-of-care measurements of tissue metabolism and its associated vasculature *in vivo*. Both oxygenated and deoxygenated hemoglobin have broadband optical absorption spectra, which have been extensively explored by us to measure vascular oxygenation (${\mathrm{StO}}_{2}$)^23^^,^^24^ and firmly validated with the gold standard, that is, ${\mathrm{pO}}_{2}$.^25^^,^^26^ Autofluorescence of reduced nicotinamide adenine dinucleotide (NADH) and flavin adenine dinucleotide (FAD) has been explored to report the reduction-oxidation (redox) state of cells^27^ by looking at the ratio of the two (FAD/NADH) and then providing an indirect measure of the balance between glycolysis and OXPHOS. FAD and NADH-based label-free autofluorescence techniques (redox ratio, validated by Seahorse Assay^27^) have been explored by others to study cancer therapeutics.^28^ Label-free Raman techniques have also been explored extensively to report tumor metabolism.^29^^,^^30^ Though these two label-free techniques are promising for tumor metabolism studies, they are primarily explored for diagnostic studies or therapy response predictions.^28^^,^^29^ Alternatively, we have exploited a metabolic probes-based approach to measure tissue glycolysis and mitochondrial function directly and explicitly on small animals *in vivo*.^23^ The 2-[N-(7-nitrobenz-2-oxa-1, 3-diazol-4-yl) amino]-2-deoxy-d-glucose (2-NBDG) has been extensively used in cell and animal models to quantify glycolysis.^23^^,^^31^ Although 2-NBDG does not provide insights into the entire glycolytic pathway, it measures glucose uptake analogous to clinically accepted FDG-PET imaging.^32^^,^^33^ TMRE has been utilized extensively to measure mitochondrial membrane potential (MMP) to study OXPHOS.^34^^–^^36^

Though the measurement of each endpoint is well established by us,^36^ it will be critical to integrate all these measurements into one single highly portable device to provide rapid quantification of these endpoints simultaneously on the same tissue site, which allows one to perform multi-dimensional metabolic analysis^37^^,^^38^ on tumors that may provide more insights into cancer biology. Here, we report a novel PEERS (portable, easy-to-use, easy-to-access, rapid, systematic) optical spectroscopy for metabolic characterizations on biological models *in vivo* to enable scientific discoveries to translate more efficiently to clinical interventions. Specifically, we developed (1) a highly portable and low-cost optical spectroscopy platform with a tumor-sensitive fiber probe for diffuse reflectance and fluorescence measurements on biological tissues and (2) novel easy-to-use spectroscopic algorithms for rapid quantification of metabolic parameters on biological tissues *in vivo*. To demonstrate the proof-of-concept of our reported PEERS optical spectroscopy techniques, we performed tissue-mimicking phantom studies, human subject pilot tests, and *in vivo* animal studies for rapid quantification of vascular and metabolic parameters. We also validated the easy-to-use algorithms with our previously reported Monte Carlo (MC) techniques^23^ for accurate and rapid spectroscopy data processing. Our tissue-mimicking phantoms, human subjects, and *in vivo* animal studies showed that our optical spectroscopy along with the novel spectroscopic algorithms could quantify the major metabolic and vascular parameters on biological tissues with high accuracy. Our *in vivo* animal studies also captured the highly diverse metabolic and vascular phenotypes of head and neck tumors with different radiation sensitivities. Our reported novel optical spectroscopy will provide a new way (PEERS: point-of-care, easy-to-use, easy-to-access, rapid, systematic) for characterizing tumor metabolism and its vascular microenvironment *in vivo*, and it will have a broad impact across many biomedical fields through the lens of tissue metabolism and vascular microenvironment.

## 2 Materials and Methods

### 2.1 Portable multi-Parametric Optical Spectroscopy Platform

**Fig. 1:**
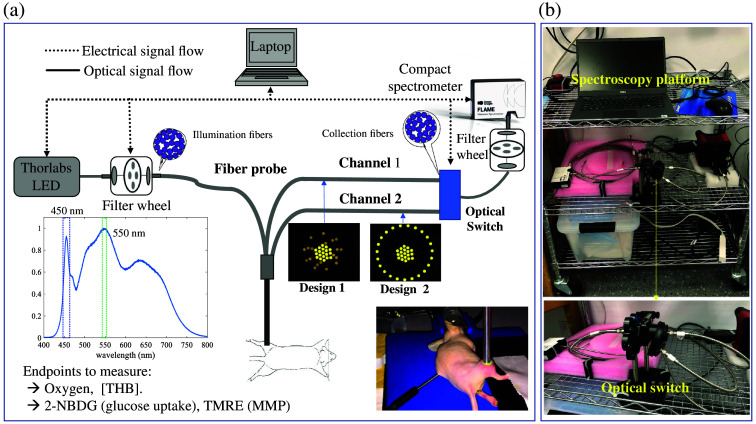
(a) Schematic of the portable optical spectroscopy platform with a custom-designed tumor-sensitive dual channel fiber probe. (b) Photo of the portable optical spectroscopy platform and the custom-designed optical switch. The total cost of the major optical components of the system is ∼10  k USD.

To minimize the system size and cost, we have identified a Solis™ High-Power white LED source (SOLIS-3C, Thorlabs) for both fluorescence and diffuse reflectance measurements. As illustrated in Fig. 1(a), a 450-nm bandpass filter (±12.5  nm) was used to generate light for 2-NBDG (glucose uptake probe) excitation, and a 550-nm bandpass filter (±12.5  nm) was used to generate light for TMRE (MMP probe) excitation. A neutral density filter (ND = 2.0, Thorlabs) was used for diffuse reflectance illumination to protect the spectrometer from over-exposure. A custom-designed fiber optics probe (LEONI Fiber) with two groups of unique source–detector distances was designed based on our former numerical studies^39^ to enable tumor-sensitive metabolic characterizations on small tumor models. A total of 19 illumination fibers were bundled in the center of the common end for light delivery, whereas a total of 19 fibers were used for channel 1 light collection, and a total of 22 fibers were used for channel 2 light collection. The diameter of each fiber is 200  μm. The average source–detector separation for channel 1 was 1.5 mm, whereas the average source–detector separation for channel 2 was 3 mm. The max illumination power from the illumination channel can reach up to 2.0 mW at 450 nm and 1.5 mW at 550 nm, respectively. In the collection end, a custom-designed optical switch [shown in Fig. 1(b), 2 channels to 1 channel] was used to ensure the signal from the two collection channels can be acquired sequentially by a compact spectrometer (FLAME-T-VIS-NIR, Ocean Optics). Two long-pass filters (515 nm for 2-NBDG, and 575 nm for TMRE) were installed in the optical switch for fluorescence measurement to remove excitation light, whereas no filter was used for diffuse reflectance collection. Once the basic operation was established, the system was packaged into a small cart [Fig. 1(b)] for future point-of-care measurements.

### 2.2 Monte Carlo Model and Empirical Methods for Vascular and Metabolic Parameters Estimation

Our formerly reported MC inverse model^23^ was adapted with our new spectroscopy system for both diffuse reflectance and fluorescence data processing.^40^^,^^41^ Briefly, the diffuse reflectance MC inversion model adaptively fits the measured reflectance spectrum to the simulated spectra until the sum of squares error between the two is minimized, whereas the fluorescence MC inversion model extracts the intrinsic fluorescence with the implementation of absorption and scattering information extracted from the reflectance inversion model.^40^ The MC-extracted absorption spectra were further fitted with a linear combination of the extinction spectra of oxy-hemoglobin and deoxyhemoglobin to quantify tissue vascular saturation (${\mathrm{StO}}_{2}$) and total hemoglobin concentration ([THB]).^42^ The MC model processed fluorescence spectra were used to report the key metabolic parameters with the use of fluorescence probes.

To enable rapid and easy quantification of optical metabolic and vascular parameters, we also explored ratio-metric and equation-based analytical methods for spectral data processing. Several diffuse reflectance ratios comprised of isosbestic wavelengths have been explored to estimate [THB],^43^ whereas the diffuse reflectance ratio at 584 and 545 nm with a narrower bandwidth provided a more sensitive measurement of [THB].^43^ Therefore, the ratio of diffuse reflectance at 584 nm and 545 nm was explored to indicate [THB] in our study. The corrected absorbance equation ^44^ has been explored to estimate the tissue vascular ${\mathrm{StO}}_{2}$ with the use of proper wavelength bands. Specifically, the corrected absorbance [A(λ)] can be calculated from diffuse reflectance [R(λ)] by Eq. (1) to remove the scattering distortion,

$$A(\lambda )=\log(\frac{1}{R(\lambda )})-(a+m\times \lambda ),$$ (1)

where the a and m are the intercept and slope of the least square fitted line that can be estimated using reflectance at the scattering domain band (620 to 680 nm). The corrected absorbance can also be expressed by Eq. (2)

$$A(\lambda )=C_{\mathrm{Hb}}\times \varepsilon_{\mathrm{Hb}}(\lambda )\times \langle L\rangle +C_{\mathrm{HbO}2}\times \varepsilon_{\mathrm{HbO}2}(\lambda )\times \langle L\rangle ,$$ (2)

where the A(λ) is a sum of absorptions contributed by the oxygenated hemoglobin and deoxygenated hemoglobin, $\varepsilon_{\mathrm{Hb}}(\lambda )$ and$\varepsilon_{\mathrm{HbO}2}(\lambda )$ are the molecular extinction coefficient of deoxyhemoglobin and oxyhemoglobin^42^ (knowns), and the ⟨L⟩ is the average optical path length (unknown). ${\mathrm{StO}}_{2}$ can then be easily estimated based on the concentrations ($C_{\mathrm{HbO}2\;}$ and $C_{\mathrm{Hb}\;}$) of the two molecules using Eq. (3) even though the ⟨L⟩ is unknown:

$${\mathrm{StO}}_{2}=\frac{C_{\mathrm{HbO}2}*\langle L\rangle }{C_{\mathrm{Hb}}\times \langle L\rangle +C_{\mathrm{HbO}2}\times \langle L\rangle }=\frac{C_{\mathrm{HbO}2}}{C_{\mathrm{Hb}}+C_{\mathrm{HbO}2}}.$$ (3)

To estimate $C_{\mathrm{HbO}2}$ and $C_{\mathrm{Hb}\;}$, at least two wavelengths are required. The wavelengths of 555 and 575 nm were used in our study for ${\mathrm{StO}}_{2}$ estimation, but other non-isosbestic wavelengths could also be used for the same purpose.^44^

We have reported a novel ratio-metric method as illustrated by Eq. (4) for rapid extraction of intrinsic fluorescence by dividing the fluorescence spectra by diffuse reflectance intensities recorded at excitation and emission peaks with a pair of system-dependent power (α,β):^45^

$$F_{\mathrm{corr}}(\lambda )=\frac{F_{\mathrm{raw}}(\lambda )}{[R_{\mathrm{ex}}{]}^{\alpha }[R_{\mathrm{em}}{]}^{\beta }}.$$ (4)

Relying on this foundation, we further explored the ratio-metric method for intrinsic fluorescence extraction from fluorescence measured from biological tissues using our new optical spectroscopy platform. The MC inversion model served as a benchtop reference to validate the proposed easy-to-use ratio-metric and analytical methods for metabolic and vascular parameter quantifications.

### 2.3 Tissue-Mimicking Phantoms and Human Subject Pilot Test

Tissue-mimicking phantom studies were used to verify the optical spectroscopy system along with various spectroscopic data processing methods for accurate quantification of the intrinsic fluorescence signal, absorption, and scattering as described previously.^25^^,^^45^^–^^47^ These phantoms were also used to validate the ratio-metric techniques and equation-based analytical methods against the MC model for accurate quantification of the key metabolic and vascular parameters. Careful validation of the optical platform and data processing methods using proper tissue-mimicking phantoms is necessary to ensure the new techniques are applicable to the range of conditions under which they will be deployed. To ensure the tissue-mimicking phantoms cover the optical properties of biological tissue samples in this study, the optical properties of the phantoms were designed based on the optical properties of mice flank tumor models.^41^ Turbid medium phantoms (a mixture of DI water, polystyrene spheres, and human hemoglobin) with various reduced scattering and absorption levels^41^ were created to characterize the system for accurate measurement of absorption and scattering. Two groups of tissue-mimicking phantoms with different initial reduced scattering levels (10 and $20\;{\mathrm{cm}}^{-1}$ on average between 400 and 600 nm) were prepared. Within each group of phantoms, seven increasing concentrations of hemoglobin were added to generate average absorption coefficients of 1.0 to $7.5\;{\mathrm{cm}}^{-1}$ (on average between 400 and 600 nm). Hemoglobin concentration was increased by adding aliquots of the stock hemoglobin solution with a known absorption coefficient spectrum that was determined by a spectrophotometer. After each addition of hemoglobin stock solution, diffuse reflectance spectra were measured from the phantom. On the other hand, tissue-mimicking fluorescence phantoms with a 2-NBDG concentration of 2 to 6  μM, and a TMRE concentration ranging from 30 to 90 nM were created to characterize the system for accurate measure of intrinsic fluorescence signals for 2-NBDG and TMRE at biologically relevant concentrations.^45^ The tissue-mimicking fluorescence phantoms had the following average absorption coefficients and reduced scattering coefficients (400 to 600 nm): $\mu_{a}$ = [1.5, 3.0, 4.5] ${\mathrm{cm}}^{-1}$ and $\mu_{s}^{\prime }$ = [10, 20] ${\mathrm{cm}}^{-1}$ that will cover the mice skin or flank tumor optical properties.^45^ The tissue-mimicking fluorescence phantoms were also used to identify the system-dependent power values (α,β) for *in vivo* biological tissue fluorescence data correction. The optimal parameter set for the pair of powers [α, β] are the ones that could converge the fluorescence spectra from different absorption-scattering combinations, but the same fluorophore concentration for the selected correction wavelengths. The nonlinear multivariable optimization function, fminsearch from the Matlab optimization toolbox, was used to find the optimal parameter set [α, β]. This procedure has been detailed in our former publication but uses a different optical spectroscopy platform in purely phantom studies.^45^ To evaluate the equation-based analytical method for rapid quantification of tissue ${\mathrm{StO}}_{2}$ *in vivo*, human-subject pilot tests were also conducted. Specifically, four healthy volunteers’ fingertips were measured using the proposed optical spectroscopy to report the tissue ${\mathrm{StO}}_{2}$_._ Five measurements on each subject’s fingertips were conducted to get the average tissue ${\mathrm{StO}}_{2}$ for each subject.

### 2.4 Animal Study

To demonstrate the feasibility of the optical spectroscopy system for *in vivo* measurements of tumor metabolism and vasculature, a pilot test on a total of 14 mice was conducted according to a protocol approved by the University of Kentucky Institutional Animal Care and Use Committee (IACUC). Male or female athymic nude mice (nu/nu, Jackson Laboratory) with an age of 8 to 10 weeks were used for these studies. All mice were housed in an on-site housing facility with *ad libitum* access to food and water and standard 12-h light/dark cycles. Animals were assigned to a (1) radio-resistant tumor group (rSCC-61, n=7) and (2) radio-sensitive tumor group (SCC-61, n=7). Mice assigned to tumor groups received a subcutaneous injection of rSCC-61 or SCC-61 cells ($1\times 10^{6}$ to $2\times 10^{6}$ cells in 100  μL PBS with Matrigel) in the flank under anesthesia with inhaled isoflurane (1% to 2% v/v) in room air. The radio-resistant cells (rSCC-61) were generated by introducing multiple courses of low-dose RT on their parental radio-sensitive cells (SCC-61).^48^^,^^49^ After the tumor cell injection, mice were returned to the cage and monitored for 4 weeks. On day 10 after the tumor injection or the tumor diameter reached ∼7  mm, the tumors were characterized using the quantitative optical spectroscopy platform under isoflurane anesthesia.

### 2.5 Optical Measurements and Data Analysis

All tissue-mimicking phantoms were measured using the optical spectroscopy introduced in Fig. 1. Diffuse reflectance spectra (integration time: 8 ms) were acquired from 450 to 650 nm. Fluorescence spectra (integration time: 800 ms) were acquired from 520 to 600 nm for 2-NBDG and from 565 to 650 nm for TMRE. In human-subject pilot tests, only diffuse reflectance measurements were taken using the same optical configurations. In animal studies, all mice were fasted for 6 h and anesthetized with 1% to 2% v/v isoflurane for optical spectroscopy study. Optical measurements on small animals were conducted using the exact same optical configurations that are used in tissue-mimicking phantom measurements so one can directly use the phantom studies’ established models to process the animal data. All mice received a tail-vein injection of TMRE (100  μL of 100  μM) first and then a tail-vein injection of 2-NBDG (100  μL of 6 mM 2-NBDG) with a 20-min delay.^23^ Prior to any injection, baseline diffuse reflectance and fluorescence spectra were measured from the tissue. Optical measurements were obtained by placing the fiber probe gently on the tumors. All measurements on each mouse were acquired continuously for a period of 80 min in a dark room.

All diffuse reflectance and fluorescence spectra were calibrated using a 20% reflectance standard (Spectralon, Labsphere) and a fluorescence standard (USF 210-010, LabSphere), respectively. Both MC models and the easy-to-use analytical methods were used for spectral data processing to estimate the relevant vascular and metabolic endpoints. The extracted optical parameters from phantom studies were compared with their corresponding true values. The processed diffuse reflectance spectra from human subjects or mice were used to estimate vascular parameters using either MC models or equation-based analytical methods. The fluorescence spectra from mice were processed using either the MC model or equation-based analytical methods to estimate metabolic parameters. The 2-NBDG and TMRE kinetic uptake curves for the mice were created from the mean data for the peak band (emission peak wavelength ±10  nm) based on the fluorescent spectra taken at different time points. Because of the 20-min delay between TMRE injection and 2-NBDG injection, the 2-NBDG uptake at 60 min post-2-NBDG injection ($2\text{-}{\mathrm{NBDG}}_{60}$) and the TMRE uptake at 80 min post TMRE injection (${\mathrm{TMRE}}_{80}$) that measured at the same time point were used to report final 2-NBDG and TMRE uptake. The metabolic parameters, ${\mathrm{StO}}_{2}$, and [THB] between experimental groups were compared using a Student’s t-test. A p-value less than 0.05 was statistically significant. Pearson’s correlation coefficients and p-values were calculated to assess the relationship between variables among different experimental groups. MATLAB (Mathworks, United States) was used to perform all statistical analyses.

## 3 Results

### 3.1 Ratio-Metric Method for Easy and Accurate Estimation of Total Hemoglobin Concentrations in Tissue-Mimicking Phantoms

**Fig. 2:**
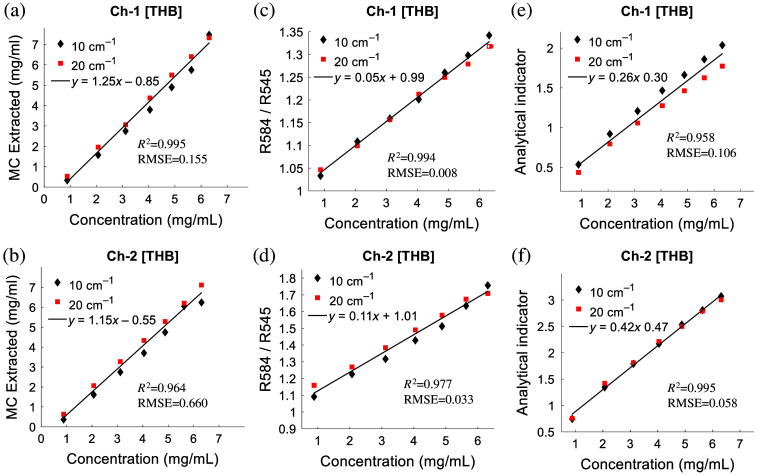
Comparison of MC model extracted and expected [THB] for channel 1 (a) and channel 2 (b); comparison of ratio-metric method extracted [THB] indicator and expected [THB] for channel 1 (c) and channel 2 (d). Comparison of analytical method extracted [THB] indicator and expected [THB] for channel 1 (e) and channel 2 (f).

Figure S1 in the Supplementary Material shows that our new optical spectroscopy along with the MC inversion model can accurately quantify the absorption and scattering properties of turbid medium. The high agreement between the MC simulated and the measured spectra shown in Figures S1(c) and S1(d) in the Supplementary Material enables the accurate estimate of absorption and scattering levels of the tissue-mimicking phantoms. Figures S1(e) and S1(f) in the Supplementary Material show the comparison between the MC extracted and the corresponding expected absorption coefficients with an average percent error of 5.7% and 5.2%, respectively, for the two channels. Figures S1(g) and S1(h) in the Supplementary Material show the comparison between the MC extracted and the expected reduced scattering coefficients with an average percent error of 4.6% and 7.1%, respectively, for the two collection channels. Based on the MC extracted absorption information, one can easily estimate the [THB] as shown in Figs. 2(a) and 2(b). As expected, the MC model can accurately estimate the [THB] from the tissue-mimicking phantoms for the two channels. Figures 2(c) and 2(d) show the performance of the simple ratio-metric method for the estimation of [THB]. As shown in Figs. 2(c) and 2(d), the ratios of diffuse reflectance at 584 nm and 545 nm can indicate the [THB] with comparable accuracy compared with the MC model using either collection channel. Figures 2(e) and 2(f) show the performance of the analytical method [Eq. (1)] for the estimation of [THB]. As illustrated, the analytical method can also accurately estimate the [THB] from the tissue-mimicking phantoms using either of the two channels.

### 3.2 Analytical Method for Easy and Accurate Estimation of Tissue Oxygen Saturation on Human Subjects and Tissue-Mimicking Phantoms

**Fig. 3:**
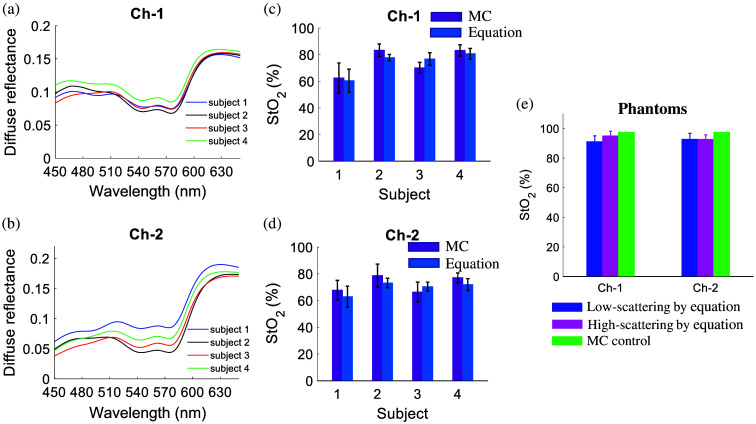
Representative measured diffuse reflectance spectra on the four human subjects’ fingertips using channel l (a) and channel 2 (b); The ${\mathrm{StO}}_{2}$ values estimated by the MC model and equation-based method for all four subjects using the spectra collected by channel l (c) and channel 2 (d). (e) The ${\mathrm{StO}}_{2}$ values estimated by the equation-based method for tissue-mimicking phantoms with various reduced scattering (10 and $20\;{\mathrm{cm}}^{-1}$) and absorption levels (1.0 and $7.5\;{\mathrm{cm}}^{-1}$).

Figures 3(a)–3(d) show representative reflectance spectra measured on volunteers’ fingertips and the corresponding ${\mathrm{StO}}_{2}$ estimated using the MC inverse model or the equation-based analytical method. Figures 3(a) and 3(b) show the representative diffuse reflectance spectra measured on the four subjects using the two collection channels, whereas Figs. 3(c) and 3(d) show the ${\mathrm{StO}}_{2}$ estimated from the diffuse reflectance spectra using the MC inverse model and equation-based method for all subjects. The ${\mathrm{StO}}_{2}$ values estimated by the equation-based methods are almost identical to the ${\mathrm{StO}}_{2}$ values estimated by the MC model for all four subjects as shown in Figs. 3(c) and 3(d). Figure 3(e) shows the analytical method estimated ${\mathrm{StO}}_{2}$ values from diffuse reflectance spectra measured on phantoms with various scattering and absorption levels [Figs. S1(a) and S1(b) in the Supplementary Material]. The results showed that ${\mathrm{StO}}_{2}$ values estimated from low-scattering phantoms are almost identical to those from high-scattering phantoms. The ${\mathrm{StO}}_{2}$ values estimated by the analytical method are comparable to those estimated by the MC model and are consistent with formerly published data from turbid phantoms with similar oxygen conditions.^50^

### 3.3 Ratio-Metric Method for Easy and Accurate Estimation of Intrinsic Fluorescence Signal in Tissue-Mimicking Phantoms

**Fig. 4:**
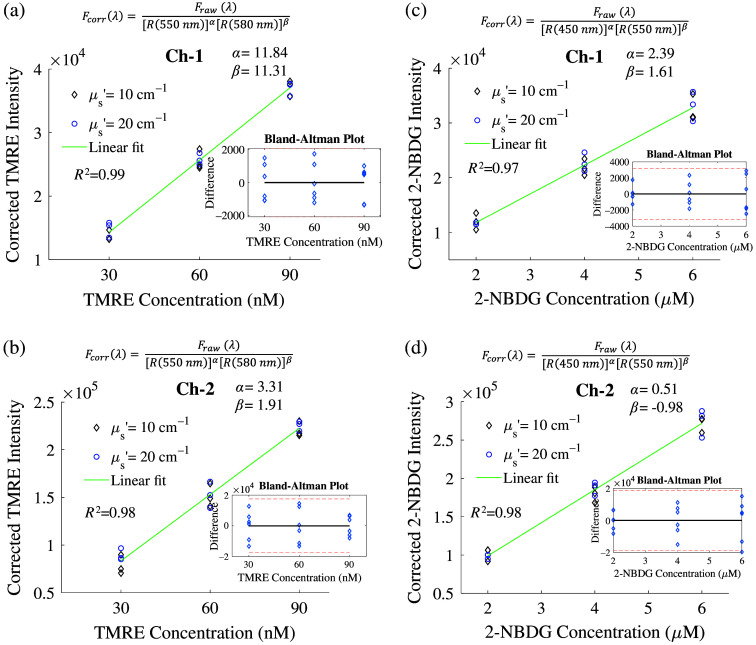
Empirical model with a pair of single wavelengths for accurate attenuation correction in TMRE and 2-NBDG signals. Comparison between the corrected fluorescence peak intensities and the true TMRE concentrations along with the corresponding Bland–Altman plot for channels 1 and 2 (a) and (b). Comparison between the corrected fluorescence peak intensities and their corresponding true 2-NBDG concentrations along with the corresponding Bland–Altman plot for channels 1 (c) and 2 (d).

Figure S2 in the Supplementary Material shows attenuation corrections for TMRE and 2-NBDG fluorescence spectra using our empirical ratio-metric model. Diffuse reflectance at the excitation source peak and the emission peak with optimized power function values were used for the fluorescence corrections. The graphs in Fig. S2 in the Supplementary Material show significant distortions on the TMRE or 2-NBDG fluorescence spectra caused by either absorption or scattering, whereas the ratio-metric method can effectively correct these distortions for both TMRE and 2-NBDG using the two collection channels. Figure 4 shows the quantitative comparison between the ratio-metric method corrected fluorescence peak intensities and true TMRE or 2-NBDG concentrations. The linear fit of the corrected fluorescence peak intensities versus the fluorescence probe concentrations yielded high coefficients of determination ($R^{2}$) indicating the high performance of the ratio-metric technique for both TMRE and 2-NBDG corrections. Specifically, an $R^{2}$ of 0.99 and 0.98 was achieved for TMRE correction using the two channels. An $R^{2}$ of 0.97 and 0.98 was achieved for 2-NBDG correction using the two channels. The p-value for all determination coefficients was less than 0.001. The power function values of [α,β] optimized from the phantom studies will be used for future *in vivo* data correction.

### 3.4 Optical Measure of Vascular Parameters of Tumors *In Vivo*

**Fig. 5:**
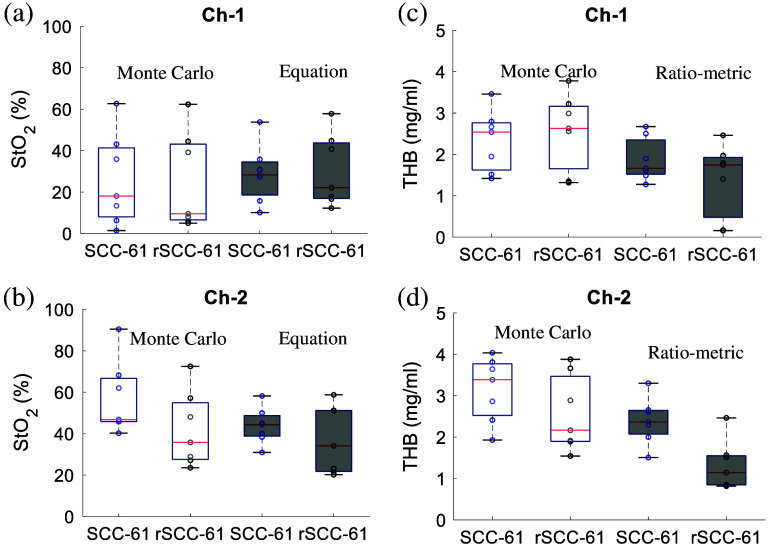
${\mathrm{StO}}_{2}$ values estimated by the MC model and equation-based method for all SCC-61 and rSCC-61 tumors using reflectance spectra collected by channel 1 (a) and channel 2 (b). The [THB] values estimated by the MC model and ratio-metric method for all SCC-61 and rSCC-61 tumors using reflectance spectra collected by channel 1 (c) and channel 2 (d).

Figure 5 shows the optically measured vascular parameters for both SCC-61 and rSCC-61 tumors *in vivo*. These parameters were estimated from the diffuse reflectance spectra processed by the MC inversion model or the equation-based method. The equation-based method provided similar ${\mathrm{StO}}_{2}$ values compared to that estimated by the MC model for the two collection channels as shown in Figs. 5(a) and 5(b). Figure 5(a) shows that channel 1 measured rSCC-61 and SCC-61 tumors had comparable ${\mathrm{StO}}_{2}$ values, whereas Fig. 5(b) shows that channel 2 measured rSCC-61 tumors had lower ${\mathrm{StO}}_{2}$ values (not statistically significant) compared to SCC-61 tumors. Figures 5(c) and 5(d) show [THB] of head and neck tumors estimated by the MC model or the ratio-metric method. Generally, the ratio-metric method yielded lower [THB] values compared with that estimated by the MC model, whereas the trends of [THB] between the two tumor lines characterized by the two techniques are the same. Specifically, channel 1 measured rSCC-61 tumors had comparable [THB] compared with that in SCC-61 tumors, whereas channel 2 measured rSCC-61 tumors had lower [THB] compared with SCC-61 tumors.

### 3.5 Optical Measure of Metabolic Parameters of Tumors *In Vivo*

Figure S3 in the Supplementary Material shows the 2-NBDG uptake kinetic profiles and TMRE uptake kinetic profiles characterized using the raw fluorescence spectra, MC model processed fluorescence spectra, and the ratio-metric method processed fluorescence spectra. The top panel of Fig. S3 in the Supplementary Material shows that rSCC-61 tumors had significantly different 2-NBDG kinetic profiles and intensities, but similar TMRE kinetic profiles and intensities when the raw fluorescence data was used. The middle panel of Fig. S3 in the Supplementary Material shows the MC model was able to remove the distortions on the fluorescence signals, thereby highlighting the 2-NBDG and TMRE differences between rSCC-61 tumors and SCC-61 tumors. The bottom panel of Fig. S3 in the Supplementary Material shows the ratio-metric model can also correct the fluorescence distortions and enhance the 2-NBDG and TMRE differences between rSCC-61 tumors and SCC-61 tumors.

**Fig. 6:**
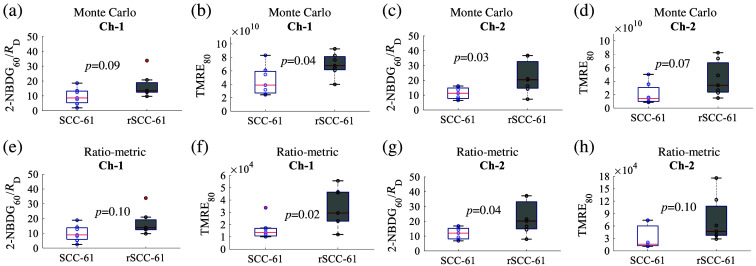
Delivery corrected 2-NBDG60 uptake and TMRE80 uptake quantified from the MC model processed fluorescence on head and neck tumors measured by the two channels (a)–(d). Delivery corrected 2-NBDG60 uptake and TMRE80 uptake quantified from the ratio-metric method processed fluorescence on tumors measured by the two channels (e)–(h). Comparison of the mean intensity of $2\text{-}{\mathrm{NBDG}}_{60}/R_{D}$ and TMRE80 across animal groups was performed with two sample t-tests using the MATLAB (Mathworks, United States) statistics toolbox. Delivery rate ($R_{D}$) is defined as 2-NBDGpeak/Timepeak as reported previously.^51^

Figure 6 shows delivery corrected $2\text{-}{\mathrm{NBDG}}_{60}$ uptake^51^ and ${\mathrm{TMRE}}_{80}$ uptake in rSCC-61 tumors and SCC-61 tumors. These metabolic parameters were estimated from the fluorescence spectra processed by the MC inversion model or the ratio-metric method. The trends of the two metabolic parameters between the two tumor lines characterized by the two data processing techniques are the same. Figures 6(a) and 6(e) show that channel 1 measured rSCC-61 tumors had higher $2\text{-}{\mathrm{NBDG}}_{60}/R_{D}$ (not statistically significant) compared to SCC-61 tumors, whereas Figs. 6(b) and 6(f) show that channel 1 measured rSCC-61 tumors had significantly higher ${\mathrm{TMRE}}_{80}$ uptake (p<0.05) compared with SCC-61 tumors. Figures 6(c) and 6(g) show that channel 2 measured rSCC-61 tumors had significantly higher $2\text{-}{\mathrm{NBDG}}_{60}/R_{D}$ (p<0.05) compared with SCC-61 tumors, whereas Figs. 6(d) and 6(h) show that channel 2 measured rSCC-61 tumors had higher ${\mathrm{TMRE}}_{80}$ uptake (not statistically significant) compared with SCC-61 tumors.

### 3.6 Multiple-Dimensional Data Analysis Provides New Information for Cancer Biology

**Fig. 7:**
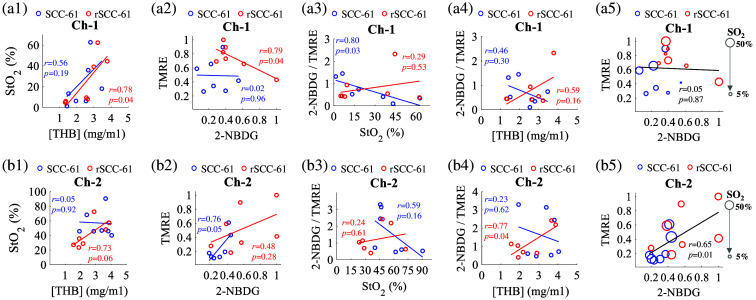
Correlations between ${\mathrm{StO}}_{2}$ and [THB] for two types of tumors for channel 1 (a1) and channel 2 (b1). Correlations between delivery corrected 2-NBDG uptake and TMRE uptake for two types of tumors for channel 1 (a2) and channel 2 (b2). Correlations between metabolic ratio and ${\mathrm{StO}}_{2}$ for two types of tumors for channel 1 (a3) and channel 2 (b3). Correlations between metabolic ratio and [THB] for two types of tumors for channel 1 (a4) and channel 2 (b4). Scatter plots of metabolic endpoints along with baseline ${\mathrm{StO}}_{2}$ for all tumors in one graph for channel 1 (a5) and channel 2 (b5). Baseline ${\mathrm{SO}}_{2}$ levels are represented by marker size. Larger markers indicate an increased ${\mathrm{SO}}_{2}$. All metabolic values were normalized to their own global highest value, that is, the highest $2\text{-}{\mathrm{NBDG}}_{60}/R_{D}$ and ${\mathrm{TMRE}}_{80}$ intensity across all tumors. Pearson’s correlation coefficients and p-values were calculated using the MATLAB (Mathworks, United States) statistics toolbox.

Simultaneous measurement of several vascular and metabolic endpoints on the same tumor site allows us to investigate the potential relationship between tumor metabolism and the associated vasculature. Figure 7 shows scatter plots of the relationship between different combinations of the four functional endpoints measured using optical spectroscopy. All the data shown here are processed by the MC model. However, it should be noted that the ratio-metric processed data points also yielded similar trends for all data shown in Fig. 7. All the $2\text{-}{\mathrm{NBDG}}_{60}/R_{D}$ and ${\mathrm{TMRE}}_{80}$ values were normalized to the highest global value, that is, the highest $2\text{-}{\mathrm{NBDG}}_{60}/R_{D}$ and ${\mathrm{TMRE}}_{80}$ across all tumors. The top panel of Fig. 7 shows the correlations among the metabolic and vascular endpoints for head and neck tumors characterized by channel 1. Figure 7(a1) shows that channel 1 measured baseline ${\mathrm{StO}}_{2}$ levels in both two tumor lines are positively correlated with [THB], but only statistically significant for rSCC-61 tumors (r=0.78, p=0.04). Figure 7(a2) shows that the channel 1 measured $2\text{-}{\mathrm{NBDG}}_{60}/R_{D}$ was negatively correlated with ${\mathrm{TMRE}}_{80}$ (r=0.79, p=0.04) for rSCC-61 tumors, whereas it appears that there was a weak correlation between the two metabolic endpoints for SCC-61 tumors. Figure 7(a3) shows that the channel 1 characterized metabolic ratio (the ratio between $2\text{-}{\mathrm{NBDG}}_{60}/R_{D}$ and ${\mathrm{TMRE}}_{80}$) was negatively correlated with ${\mathrm{StO}}_{2}$ for SCC-61 tumors (r=0.80, p=0.03) but not for rSCC-61 tumors. Figure 7(a4) shows that channel 1 characterized metabolic ratio was not correlated with [THB] for both tumor lines. Figure 7(a5) shows scatter plots of metabolic endpoints along with baseline ${\mathrm{StO}}_{2}$ represented as different-sized symbols for all tumors. Figure 7(a5) shows that SCC-61 tumors with lower ${\mathrm{StO}}_{2}$ tend to have lower ${\mathrm{TMRE}}_{80}$ but higher $2\text{-}{\mathrm{NBDG}}_{60}/R_{D}$, whereas no clear trend between the metabolic endpoint and ${\mathrm{StO}}_{2}$ was observed for rSCC-61 tumors.

The bottom panel of Fig. 7 shows correlations for head and neck tumors characterized by channel 2. Figure 7(b1) shows that channel 2 measured baseline ${\mathrm{StO}}_{2}$ levels in rSCC-61 tumors are positively correlated with [THB] (r=0.73, p=0.06), whereas it appears that there was a weak correlation between the two vascular endpoints for channel 2 measured SCC-61 tumors. In contrast, Fig. 7(b2) shows that channel 2 measured $2\text{-}{\mathrm{NBDG}}_{60}/R_{D}$ was positively correlated with ${\mathrm{TMRE}}_{80}$ (r=0.76, p=0.05) for SCC-61 tumors, whereas it appears that there was a weak correlation between the two metabolic endpoints for rSCC-61 tumors. Figure 7(b3) shows that channel 2 characterized metabolic ratio was negatively correlated with ${\mathrm{StO}}_{2}$ for SCC-61 tumors (r=0.59, p=0.16) but not for rSCC-61 tumors. Figure 7(b4) shows that the channel 2 characterized metabolic ratio was positively correlated with [THB] for rSCC-61 tumors (r=0.77, p=0.04) but not for SCC-61 tumors. Figure 7(b5) shows that there was no clear correlation between the metabolic endpoint and ${\mathrm{StO}}_{2}$ for both SCC-61 and rSCC-61 tumors, whereas it appears that TMRE uptake was positively correlated with $2\text{-}{\mathrm{NBDG}}_{60}/R_{D}$ (r=0.65, p=0.01) when we combine all metabolic data measured on the two types of tumors.

## 4 Discussion

Optical spectroscopy is well-suited for rapid *in vivo* tissue functional characterization^23^ by getting an overview of tissue status via probing a tissue volume.^52^^–^^54^ Though it lacks the capability to capture spatial heterogeneity information, optical spectroscopy is well suited for frequent and long-term longitudinal *in vivo* biomedical studies. Moreover, optical spectroscopy can provide multi-parametric measurements of tumor metabolism and its associated vasculature *in vivo*. Our previous work^23^^,^^24^ and independent studies^52^^–^^54^ showed the great potential of optical spectroscopy to provide valuable dynamic information about the metabolic status of the tissue, with important implications for different biomedical studies. However, existing quantitative spectroscopy techniques use the MC inversion model^24^ or lookup table ^54^ to extract functional parameters, neither support real-time quantification and they are highly expertise-dependent. To enable rapid optical metabolic parameters quantification and make the data processing easy to use, we reported novel spectroscopic algorithms for near real-time quantification of metabolic parameters on biological tissues *in vivo.* To maximize the ease and accessibility in obtaining *in vivo* tissue metabolism and vasculature measurements, we reported a highly portable and low-cost optical spectroscopy platform with a tumor-sensitive fiber probe for both diffuse reflectance and fluorescence measurements on biological tissues *in vivo*. Then, we performed tissue-mimicking phantom studies, human subject pilot tests, and *in vivo* animal studies to demonstrate the capability of our techniques for rapid quantification of vascular and metabolic parameters.

To minimize the spectroscopy system size and cost, we have used a Solis™ high-power white LED source (SOLIS-3C, Thorlabs) for both fluorescence and diffuse reflectance measurements. Our studies showed that the proposed portable optical spectroscopy system has sufficient sensitivity for both diffuse reflectance and fluorescence measurements. Due to the use of the new LED light source and compact spectrometer, the cost and size of our optical spectroscopy platform has been significantly reduced compared with our previously reported spectroscopy platforms.^23^ A custom-designed fiber optics probe with two groups of unique source–detector distances was implemented with our optical spectroscopy platform. The average source–detector separation for the two designs was 1.5 and 3 mm, respectively. This unique design will provide some level of flexibility in optical sensing depth for tumor biology study. The channel with a smaller source–detector separation can be used to detect shallow tumors such as epithelial cancers, whereas the channel with a larger source–detector separation may be used to detect slightly deeper tumors such as flank tumors. In the current study, the two channels were tested on the flank tumors to probe the different regions of the same tumor. It is interesting to notice that the two channels have captured different vascular and metabolic parameters of the same tumors, which suggests the volume heterogeneity of tumor vasculature and metabolism.

Our tissue-mimicking phantom studies demonstrated that our ratio-metric methods can accurately quantify [THB] and intrinsic fluorescence signals with similar accuracy compared with the MC inversion model. Our tissue-mimicking phantoms cover a wide range of absorption and scattering levels that will ensure our techniques will be applicable to various biological models that have optical properties within this range. The fluorescence phantoms with biologically relevant optical properties were used to determine the system-dependent power values for fluorescence data processing. Given these power values are system-dependent, we believe new phantom studies may not be needed for other tumor models using the same optical spectroscopy platform. However, if new samples’ optical properties are significantly away from the tissue phantoms covered range, new phantom studies with comparable tissue optical properties to the target sample are always encouraged to ensure the best accuracy for fluorescence correction.

MC model has been explored as the most accurate technique for the estimation of tissue absorption and scattering coefficients and intrinsic fluorescence from diffuse reflectance and fluorescence spectra. Then, one can easily quantify tissue vascular and metabolic parameters from the absorption and intrinsic fluorescence measurements. However, it is difficult to adapt the MC technique for real-time data processing due to the time-consuming fitting processing. Moreover, the MC technique is relatively expertise dependent which makes it challenging for users who do not have any relevant background. To address these challenges, here we demonstrated the ratio-metric or analytical methods for rapid estimation of tissue vascular parameters ([THB] and ${\mathrm{StO}}_{2}$) and intrinsic fluorescence of metabolic probes. Due to the ratio-metric or equation-based nature, our techniques will be easy to use with minimal expertise requirements and can facilitate near real-time data processing for future translational applications. However, it should be noted that the easy-to-use ratio-metric or analytical techniques have some limitations including the incapability of estimating absorption and scattering properties of tissue samples, requiring proper system calibrations with tissue-mimicking phantoms to ensure high accuracy.

To use the ratio-metric method for rapid estimation of [THB] in biological tissues, a calibration between the diffuse reflectance ratios and true [THB] has been established using tissue-mimicking phantoms. Specifically, two groups of tissue-mimicking phantoms with two initial reduced scattering levels (10 and $20\;{\mathrm{cm}}^{-1}$) were used for this calibration purpose. Within each group of phantoms, seven increasing concentrations of hemoglobin were added to generate hemoglobin concentrations from 0.9 to 6.3 mg/mL. These 14 phantoms were used to generate the calibration curves for all subsequent tissue data processing for the rapid estimation of [THB]. Given our phantoms cover a wide range of absorption and scattering levels, we believe that the calibration curve generated in Fig. 2 will have great robustness for estimating [THB] in biological tissues as demonstrated in this study. However, the presence of other tissue components (such as other types of tissue absorbers) may affect the performance of the ratio-metric method for rapid estimation of [THB] using the calibration curves determined in this study, which will be explored in our future study using phantoms with multiple types of absorbers.

To validate the analytical method for rapid and accurate ${\mathrm{StO}}_{2}$ estimation, we conducted both tissue-mimicking phantom studies and pilot human subject tests. We were not able to measure the true ${\mathrm{StO}}_{2}$ values in the tissue phantoms or human subject tissues due to the lack of resources, we can still validate the analytical method against the MC model as the MC model has been well validated by ${\mathrm{pO}}_{2}$ as reported before.^25^ Our pilot human subject tests showed that the equation-based spectroscopic technique had comparable accuracy compared with the well-established MC model for ${\mathrm{StO}}_{2}$ estimation, whereas the equation-based spectroscopic method will be less expertise-dependent and near real-time. The ${\mathrm{StO}}_{2}$ values of human subject fingertips measured by us ranged from 60% to 80%, which are consistent with previously published human subject data^55^^,^^56^ using similar optical spectroscopy techniques. Our preclinical *in vivo* animal studies using a matched model of radiation resistance for head and neck tumors further demonstrated that our new spectroscopic algorithms could quantify the key metabolic and vascular parameters of *in vivo* tumors rapidly and accurately. Our studies captured different functional endpoints of the radio-resistant and radiosensitive head and neck tumors, which suggested that both vasculature and metabolism changes are highly associated with radiation resistance development in head and neck tumors. All these studies demonstrated that our proposed spectroscopic algorithms may potentially offer new ways (easy-to-use, rapid) for optical spectroscopic data processing to quantify the key metabolic and vascular parameters.

Our *in vivo* animal studies showed that channel 1 probed vascular parameters (shallow region) are comparable between SCC-61 and rSCC-61 tumors, whereas the channel 2 probed vascular parameters (deeper region) are different between the two tumor lines. Specifically, the rSCC-61 tumors measured by channel 2 had lower ${\mathrm{StO}}_{2}$ and lower [THB] compared with SCC-61 tumors as shown in Fig. 5. This interesting phenomenon suggested that the shallow region of the two tumors may have a similar vascular microenvironment, whereas the deeper region of rSCC-61 tumors has heavier hypoxia and less blood supply compared with that of SCC-61 tumors. We also observed the high diversity of the vascular parameters for two tumor types, suggesting the high heterogeneity of the tumor vascular microenvironment. The metabolic parameters quantified by both two channels showed that rSCC-61 and SCC-61 tumors had different metabolic phenotypes. Specifically, rSCC-61 tumors had both increased $2\text{-}{\mathrm{NBDG}}_{60}/R_{D}$ and ${\mathrm{TMRE}}_{80}$ compared with SCC-61 tumors as reported in Fig. 6. Former *in vitro* cell studies showed that rSCC-61 cells had higher glucose uptake while decreased mitochondrial activities compared with SCC-61 cells.^49^ This different mitochondrial functionality between our *in vivo* studies and former *in vitro* cells might be due to the inherent different tumor microenvironments. Both increased glycolytic and mitochondrial activities in rSCC-61 tumors suggested that the radio-resistant head and neck tumors might be metabolically adaptable.^57^ It has been well-explored that the radiotherapy failure might be primarily attributed to hypoxia.^58^^,^^59^ However, increasing evidence including our own studies here shows that metabolic reprogramming may also be responsible for the development of radio resistance in cancers.^60^ This metabolic rewiring not only provides an unparalleled advantage to tumor cells to survive, grow, and metastasize under a hypoxic and nutrient-poor environment but also endows these cells with unlimited plasticity to adapt and escape immunosuppression and cancer treatment.^61^^,^^62^ Because both metabolism and vascular microenvironment alterations play a key role in the understanding of tumor treatment resistance and recurrence, it becomes crucially significant to create an innovative technology that can accurately track metabolic and vascular reprogramming in tumor cells for combating this intractable disease.

Simultaneous measurement of the key vascular and metabolic endpoints on the same tumor site provides us the opportunity to further explore the potential relationship between tumor metabolism and the associated vasculature. Figure 7(a1) showed that baseline ${\mathrm{StO}}_{2}$ levels in both two tumor lines are positively correlated with [THB], which is expected as the [THB] reflects the blood supply capability to the tumor region. Figure 7(a2) showed that the channel 1 measured $2\text{-}{\mathrm{NBDG}}_{60}/R_{D}$ was negatively correlated with ${\mathrm{TMRE}}_{80}$ (r=0.79, p=0.04) for rSCC-61 tumors but not for SCC-61 tumors. Figure 7(a5) showed that SCC-61 tumor shallow region with lower ${\mathrm{StO}}_{2}$ tend to have lower ${\mathrm{TMRE}}_{80}$ but higher $2\text{-}{\mathrm{NBDG}}_{60}/R_{D}$, whereas no clear trend between the metabolic endpoint and ${\mathrm{StO}}_{2}$ was observed for rSCC-61 tumors. These different correlations between the two different tumor lines further suggested the metabolic adaptability of rSCC-61 tumors compared with SCC-61 tumors. The correlations among the different parameters measured by channel 2 are slightly different compared with that measured by channel 1, which suggested that different regions of one same tumor may have different metabolic responses to various vascular micro-environments. Nevertheless, our preclinical study demonstrated the capability of our optical spectroscopy technique to provide rapid quantification of several key metabolic and vascular endpoints simultaneously (systematic) on the same tissue site, which allows us to perform multi-dimensional metabolic analysis on tumors that may provide more insights into cancer biology. In summary, our study demonstrated a novel PEERS (portable, easy-to-use, easy-to-access, rapid, systematic) optical spectroscopy for metabolic characterizations on biological models *in vivo* to enable scientific discoveries more efficiently. Our technology will have a broad impact across many biomedical fields through the lens of tissue metabolism and vascular microenvironment.

## 5 Conclusion

This work reported a highly portable optical spectroscopy platform with a tumor-sensitive fiber probe and novel easy-to-use spectroscopic algorithms for multi-parametric metabolic characterizations of biological tissues *in vivo*. We demonstrated our optical spectroscopy on tissue-mimicking phantoms, human subjects, and small *in vivo* tumor models. We also validated the easy-to-use algorithms with the Monte Carlo inversion models for accurate and rapid spectroscopic data processing. Tissue-mimicking phantom, human subjects, and *in vivo* animal studies showed that our optical spectroscopy along with the novel spectroscopic algorithms could quantify the major metabolic and vascular parameters on biological tissues with high accuracy. We also captured the highly diverse metabolic and vascular phenotypes of head and neck tumors with different radiation sensitivities. Our optical spectroscopy will provide a new way (PEERS: point-of-care, easy-to-use, easy-to-access, rapid, systematic) for characterizing *in vivo* tumor metabolism and vascular microenvironment, and it will have a broad impact across many biomedical fields.

## Supplementary Material



## Data Availability

The data presented in this study are available on request from the corresponding author.
