# Supplementary material for: Point-of-care optical spectroscopy platform and ratio-metric algorithms for rapid and systematic functional characterization of biological models in vivo
Source: J Biomed Opt. 2024 Dec 31;29(12):125002. doi: 10.1117/1.JBO.29.12.125002 (PMC11687633; doi:10.1117/1.JBO.29.12.125002)
Supplement: Supplementary file 1 [file JBO_029_125002_SD001.pdf]

Supplementary Material for

Point-of-care optical spectroscopy platform and novel ratio-metric algorithms for rapid and systematic functional characterization of biological models *in vivo*

Md Zahid Hasan, Jing Yan, Caigang Zhu*

All authors are with the Department of Biomedical Engineering, University of Kentucky, Lexington, Kentucky, USA (correspondence e-mail: caigang.zhu@uky.edu).

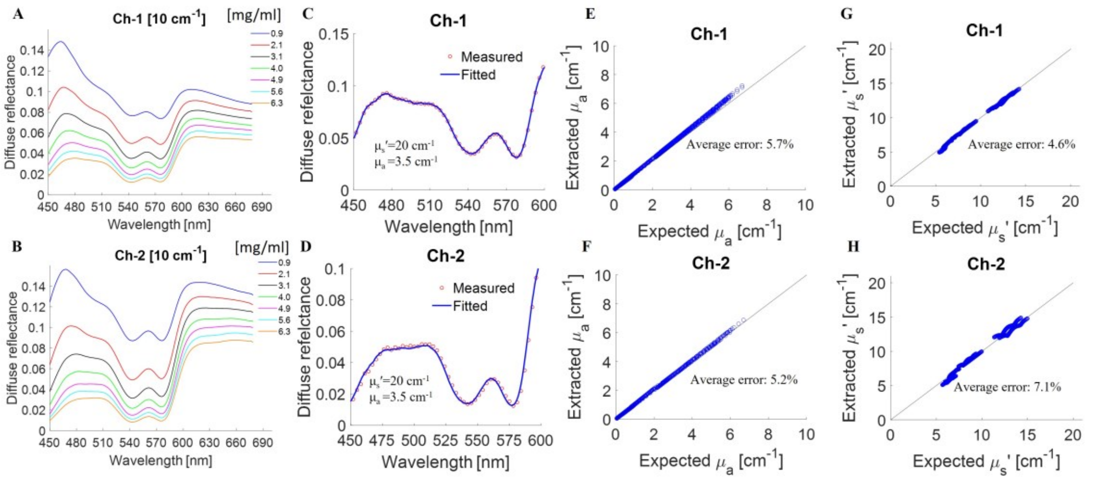

Figure S1. Measured diffuse reflectance spectra from phantoms with low scattering levels for channel 1 (A) and channel 2 (B). Representative measured reflectance spectra and MC model fitted spectra for channel 1 (C) and channel 2 (D); Comparison of optically extracted and expected absorption coefficients for channel 1 (E) and channel 2 (F); Comparison of optically extracted and expected reduced scattering coefficients for channel 1 (G) and channel 2 (H).

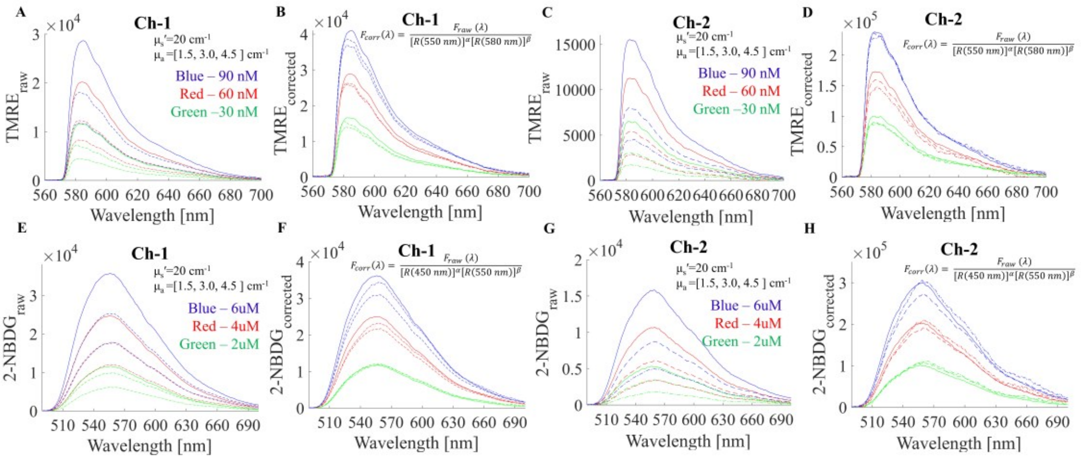

Figure S2. The empirical ratio-metric model with a pair of single wavelengths for accurate attenuation correction in TMRE and 2-NBDG phantoms. Representative raw TMRE fluorescence and corrected TMRE fluorescence spectra collected by channel 1 (A)-(B) and channel 2 (C)-(D). Representative raw

2-NBDG fluorescence and corrected 2-NBDG fluorescence spectra collected by channel 1 (E)-(F) and channel 2 (G)-(H).

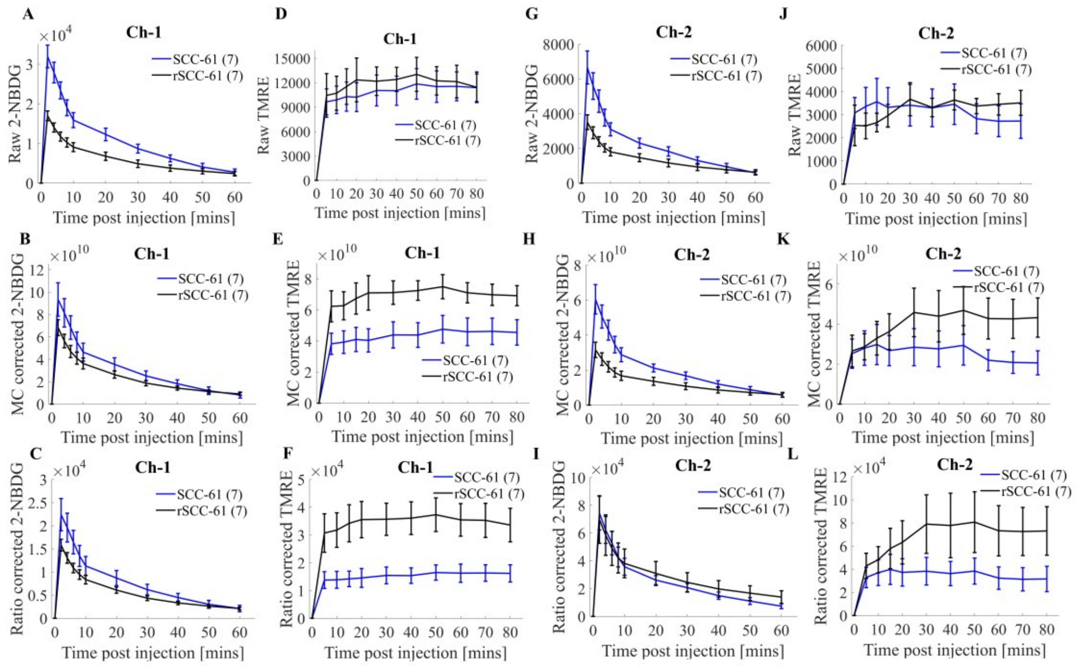

Figure S3. Representative 2-NBDG kinetic profiles obtained from raw 2-NBDG fluorescence , MC corrected 2-NBDG fluorescence , and ratio-metric method corrected 2-NBDG fluorescence measured on SCC-61 and rSCC-61 tumors using channel 1 (A)-(C) and channel 2 (G)-(I). Representative TMRE kinetic profiles obtained from raw TMRE fluorescence, MC corrected TMRE fluorescence, and ratio-metric method corrected TMRE fluorescence measured on SCC-61 and rSCC-61 tumors using channel 1 (D)-(F), and channel 2 (J)-(L).
